# A Silurian short-great-appendage arthropod

**DOI:** 10.1098/rspb.2013.2986

**Published:** 2014-03-07

**Authors:** Derek J. Siveter, Derek E. G. Briggs, David J. Siveter, Mark D. Sutton, David Legg, Sarah Joomun

**Affiliations:** 1 Earth Collections, University Museum of Natural History, Parks Road, Oxford OX1 3PW, UK; 2 Department of Earth Sciences, University of Oxford, South Parks Road, Oxford OX1 3PR, UK; 3 Department of Geology and Geophysics and Yale Peabody Museum of Natural History, Yale University, PO Box 208109, New Haven, CT 06520-8109, USA; 4 Department of Geology, University of Leicester, Leicester LE1 7RH, UK; 5 Department of Earth Sciences and Engineering, Imperial College London, London SW7 2BP, UK

**Keywords:** Arthropoda, exceptional preservation, Herefordshire Lagerstätte, Leanchoiliida, Megacheira, Silurian

## Abstract

A new arthropod, *Enalikter aphson* gen. et sp. nov., is described from the Silurian (Wenlock Series) Herefordshire Lagerstätte of the UK. It belongs to the Megacheira (=short-great-appendage group), which is recognized here, for the first time, in strata younger than mid-Cambrian age. Discovery of this new Silurian taxon allows us to identify a Devonian megacheiran representative, *Bundenbachiellus giganteus* from the Hunsrück Slate of Germany. The phylogenetic position of megacheirans is controversial: they have been interpreted as stem chelicerates, or stem euarthropods, but when *Enalikter* and *Bundenbachiellus* are added to the most comprehensive morphological database available, a stem euarthropod position is supported. *Enalikter* represents the only fully three-dimensionally preserved stem-group euarthropod, it falls in the sister clade to the crown-group euarthropods, and it provides new insights surrounding the origin and early evolution of the euarthropods. Recognition of *Enalikter* and *Bundenbachiellus* as megacheirans indicates that this major arthropod group survived for nearly 100 Myr beyond the mid-Cambrian.

## 1. Introduction

Arthropods are the most diverse invertebrates throughout the Phanerozoic. They originated in Ediacaran times, with the crown group present in lower Cambrian strata [1]. The Silurian (Wenlock Series; *ca* 425 Myr BP) Herefordshire Lagerstätte of the UK preserves invertebrates as calcitic void infills enclosed within carbonate nodules in a volcaniclastic deposit [2–4]. Since its discovery in 1994, this exceptional preservation deposit has yielded, among various invertebrates, a wide variety of remarkable arthropods that have contributed substantially to our knowledge of the palaeobiology and early history of the phylum. These include a pycnogonid [5], two synziphosurine chelicerates [6–8], a marrellomorph [9], a putative stem lineage crustacean [10], four myodocopid ostracodes [11–14], a phyllocarid [15] and a barnacle [16].

Some so-called short-great-appendage arthropods (=Megacheira [17]), such as leanchoiliids, are characterized by a first (great) head appendage with a short peduncle connected by a knuckle/elbow joint to a distal ‘claw’, the three podomeres of which each extends distally into a long flagellum [18,19]. Megacheirans have only been recorded from Cambrian deposits. Here, we describe a new genus and species of megacheiran with such a great-appendage morphology: *Enalikter aphson* from the Silurian Herefordshire fauna, representing another major arthropod group to be recognized from this Lagerstätte. Fossils from exceptionally preserved lower Palaeozoic biotas, such as the Herefordshire example, have the greatest potential for revealing the earliest stages of arthropod diversification, the stem region of the arthropod phylogenetic tree. Phylogenetic analysis of *Enalikter* and the re-evaluated Devonian taxon *Bundenbachiellus* refines the topology of this stem region, providing new insights into immediately pre-euarthropod crown-group morphologies.

## 2. Material and methods

Specimens of *Enalikter* were serially ground at 20 μm intervals. Each ground surface was captured digitally and, through using the SPIERS software suite, the resulting tomographic dataset was rendered and studied as a three-dimensional virtual fossil [20,21]. Interpretation on-screen of the virtual fossils was facilitated by variable magnification, unlimited rotational, virtual dissection and stereoscopic-viewing capabilities; they were also examined through hard-copy images.

Analysis of the phylogenetic position of *Enalikter* and *Bundenbachiellus* was performed using a modified version (see the electronic supplementary material, note S1) of the panarthropod character matrix of Legg *et al*. [22], which represents the most comprehensive morphological matrix available. The Legg *et al*. analysis included recent re-interpretations of head appendage innervation [23,24], added to which we have now also taken into account the subsequently published conclusions of Tanaka *et al*. [25]. A dataset of 314 taxa and 753 characters was analysed using maximum-parsimony in TNT v. 1.1 [26], which generated 36 most parsimonious trees (MPTs). The strict consensus tree is provided (see electronic supplementary material, figure S1), and also a summary of the topologies from the phylogenetic analyses (figure 2; electronic supplementary material, figure S2).

## 3. Systematic palaeontology

Phylum: Arthropoda von Siebold, 1848 [27].

Class: Megacheira Hou and Bergström, 1997 [17].

Order: Leanchoiliida Størmer, 1944 [28].

Family: Enaliktidae fam. nov.

Type genus: *Enalikter* gen. nov.

Other genus: *Bundenbachiellus* Broili, 1930 [29].

Family diagnosis: Leanchoiliida with subrectangular or semicircular head shield; head with three appendage pairs, the first limb uniramous with three flagella, non-geniculate between peduncle and flagella, the second and third limbs biramous; eyes absent; trunk very long and narrow, lacking paratergal folds, comprising 12 segments; trunk appendages biramous; telson with two pairs of long posterior processes/spines.

*Enalikter aphson* gen. et sp. nov.

*Etymology*: Greek, *Enalios* (of the sea) + *mastikter* (scourger), alluding to the whip-like process borne ventrally on the head; *aphares* (naked) + *soma* (body) + *gyion* (limb), referring to the exposed trunk limbs.

**Figure 1.**
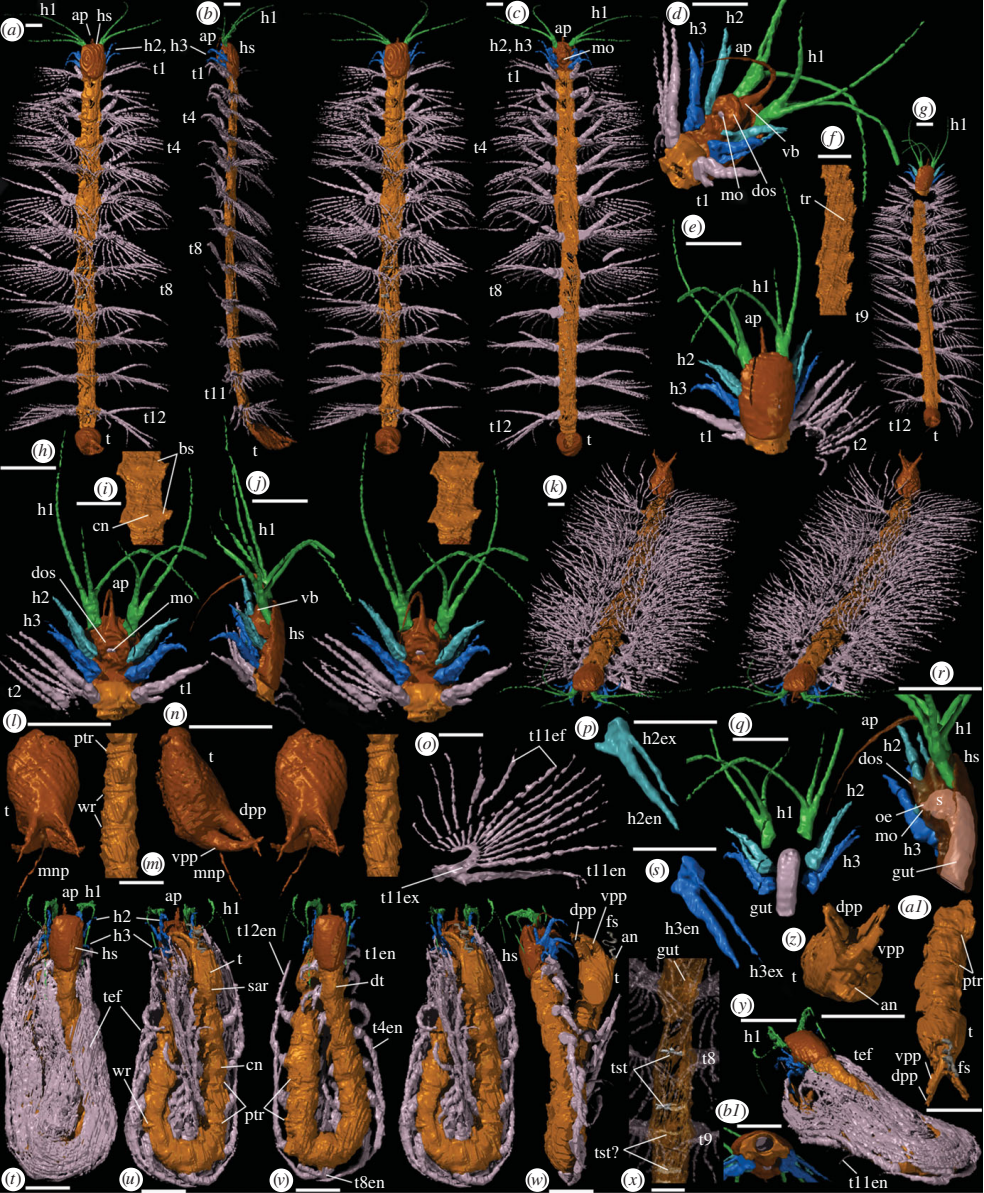
(*Opposite*.) *Enalikter aphson*, virtual reconstructions. (*a*–*c*,*k*,*o*,*x*) Holotype, OUMNH C.29631, outstretched specimen, trunk somewhat dorsoventrally compressed; (*a*–*c*,*k*) complete specimen, (*a*) dorsal stereo pair, (*b*) left lateral view, (*c*) ventral view, (*k*) anterior-oblique stereo pair, (*o*) trunk appendage 11, posteroventral view, (*x*) trunk between appendages 8 and 9, cuticle translucent, dorsal view. (*d*–*j*,*l*,*n*,*p*–*s*,*b1*) OUMNH C.29633, almost complete outstretched specimen, trunk somewhat dorsoventrally compressed, estimated length 15.9 mm; (*d*,*e*,*h*,*j*,*b1*) head and anterior-most part of trunk, (*d*) ventral posterior-oblique view, (*e*) dorsal view, (*h*) ventral stereo pair, (*i*) lateral view, (*b1*) posterior view, (*f*) trunk between trunk appendages 6 and 11, ventral view, (*g*) complete specimen, (*i*) trunk between trunk appendages 11 and 12, ventral view, (*l*,*n*) telson, (*l*) dorsal stereo-pair, (*n*) lateral view, (*p*,*s*) head appendages 2, and 3, posteroventral views, (*q*) head, with head shield and soft tissue around the gut removed, dorsal view, (*r*) head, with head shield translucent, lateral view. (*m*,*t*–*w*,*y*,*z*,*a1*) OUMNH C.29632, complete, laterally flexed specimen, estimated length 11.0 mm; (*m*) trunk between trunk appendage 10 and anterior part of telson, ventrolateral stereo pair, (*t*–*w*,*y*) complete specimen, (*t*,*v*) with exopods, and with exopods removed, dorsal stereo pair, (*u*) ventral stereo-pair, (*w*) exopods removed, lateral view, (*y*) posterior-oblique view, (*z*) telson, posterior view, (*a1*) telson and posterior part of trunk, posterodorsal view. Scale bars are all 1 mm. an, anus; ap, anterior process; bs, button-like sternite; cn, central node; dpp, dorsal posterior process; dos, disc-like oral surface; dt, dome-like tergite; fs, faecal stream; gut, midgut/intestine; h1, h2, h3, head appendages 1, 2 and 3; h2en, head appendage 2 endopod; h2ex, head appendage 2 exopod; h3en, head appendage 3 endopod; h3ex, head appendage 3 exopod; hs, head shield; mnp, medial needle-like process; mo, mouth; oe, oesophagus; ptr, prominent trunk ridge; sar, subtriangular axial region; s, stomach; t, telson; t1, t2, t4, t8, t9, t11, t12, t14, trunk appendages 1, 2, 4, 8, 9, 11, 12 and 14; t1en, trunk appendage 1 endopod; t4en, trunk appendage 4 endopod; t8en, trunk appendage 8 endopod; t11en, trunk appendage 11 endopod; t11ex, trunk appendage 11 exopod; t11ef, trunk appendage 11 exopod filaments; t12en, trunk appendage 12 endopod; tef, trunk appendage exopod filaments; tr, trunk ridge(s); tst, transverse soft tissue; tst?, transverse soft tissue?; vb, ventral boss; vpp, ventral posterior process; wr, wedge-like region.

*Holotype*: Oxford University Museum of Natural History (OUMNH C.29631) complete outstretched specimen, length 24.4 mm from anterior margin of cephalic shield to posterior margin of telson (figure 1*a*–*c*,*k*,*o*,*x*).

*Other material*: two specimens: OUMNH C.29632 and OUMNH C.29633.

Datasets from serial-grinding tomography of the specimens are housed in the Oxford University Museum of Natural History.

*Horizon and locality*: Wenlock Series, Silurian System, Herefordshire, UK.

*Other species*. None.

*Generic and specific diagnosis*. Head shield subrectangular, lacking a narrow, raised margin. Head bearing a boss-like structure ventromedially, extending anteriorly into a curved whip-like process. Trunk limb exopods with long, narrow, non-overlapping filaments lacking spines. Telson with a needle-like process medially, and two pairs of blade-like processes laterally.

*Description*. The head shield is about 1.5 times as long as wide, subrectangular in outline and dorsoventrally shallow, partially covering the first trunk segment (figure 1*e,j*). Surface sculpture is apparently lacking.

Appendage 1 originates at about 20% of the head length (figure 1*h*). It is uniramous, comprising a short peduncular section of probably two podomeres, plus three closely originating and tapering flagella (podomere numbers unresolved). One flagellum is about half as long as the other two—the ventralmost on both the best-preserved, outstretched specimens (figure 1*e,h*); an elbow/knuckle joint is lacking between peduncle and flagella. Appendage 2 is biramous and originates at about 55% of the head length. The limb base is very short, anteroposteriorly flattened, and bears a conspicuous spine-like endite. The endopod is finger-like, evenly tapered, and comprises at least three podomeres; the exopod is similar but much more slender (podomeres unresolved), and slightly shorter (figure 1*p*). Appendage 3 arises at about 85% of the head length. It is biramous and similar to appendage 2 but slightly larger, with a more robust, blunter endite; the first of the (at least four or five) podomeres of the endopod bears a median ridge (figure 1*s*).

Eyes are absent. Ventromedially, a boss-like structure (figure 1*d,h,r*) extends anteriorly into a recurved, whip-like process that is subconical proximally, more slender and tapering distally, and presumably flexible, although in all three specimens it ends beneath the mouth. The more ventral part of the boss is subcylindrical and terminates in a flat, disc-like surface with a central subcircular mouth that faces posteroventrally. A short, narrow, sediment-filled space immediately inside the mouth is interpreted as a buccal cavity and/or very short oesophagus (figure 1*r*); it connects sharply with a broader, sediment-filled cavity, interpreted as the stomach. The latter is directed dorsally before bending posteriorly in a J-shape into the intestine/midgut (figure 1*q,r,b1*).

The rest of the body, comprising a trunk and a telson, is about 14 times as long as wide. The trunk, which consists of 12 segments, is roughly parallel-sided, and is subcircular in transverse section in OUMNH C.29632 (figure 1*m,u–w,a1*), though both outstretched specimens display dorsoventral compression (see Discussion). Each tergite is dome-like (figure 1*t,v*) and lacks paratergal folds (tergopleurae). The sternite is a subcircular to subrectangular button-like structure, with a central node and a tuberculate marginal rim (figure 1*f,i,u*). At the anterior and posterior margin of each tergite and its associated sternite, there is a prominent, transverse, tuberculate ridge that encircles the trunk. In between these occur weaker, less persistent ridges (figure 1*m,u,v,a1*) representing articulations, which in places display a wedged concertina-like form, indicating segment pinching (figure 1*m,u*). These areas presumably represent arthrodial tissue, which enabled lateral flexure up to at least 90° between segments (figure 1*t–v*). Evidence of vertical trunk flexure is limited, and is at most gently upwards posteriorly (figure 1*b*). The gut is preserved discontinuously along the narrow trunk, but there is no evidence of midgut glands. Transverse, soft-tissue traces are evident posteriorly, some (? tendinous bars) coinciding with segment boundaries (figure 1*x*).

The first trunk appendage (figure 1*d,e,h*) is biramous, with a short, stout, simple limb base that lacks endites. The endopod is stenopodous, similar but larger than that of head appendage 3, with at least six or seven podomeres, the second(?) of which is raised medially. The exopod consists of a slender, tapering shaft bearing at least eight filaments (each probably from a separate podomere). The filaments are long, slender, non-overlapping and apparently suboval in section; the most proximal is the stoutest, and they become shorter distally. Trunk segments 2–12 each bear a biramous appendage pair similar to the first trunk appendage (figure 1*a–c*). Some endopods preserve two slender spinose/setal terminal projections, which were presumably present on all trunk limbs. The exopods are recurved dorsomedially in both outstretched specimens. They preserve from 11 to 17 filaments (see figure 1*o* for a typical biramous limb). These filaments are long enough to overlap at least partially those of the following appendage (figure 1*b*). The trunk appendages increase in size from the first to about the fifth, and are similar in length on successive segments (figure 1*a,g*). The endopods of the more posterior trunk appendages are slightly more slender.

The telson is ovoid in dorsal view (figure 1*a,l*) and about 1.3 times as long (medially) as wide; in lateral view, it is wedge-like, increasing in height posteriorly (figure 1*b,n,w*). Ventrally, a slightly raised, posteriorly narrowing subtriangular axial region is bounded by a very weak abaxially convex furrow (figure 1*u*). A narrow, prominent tuberculate ridge and parallel furrow, similar to those on the trunk segments, encircle the anterior margin of the telson. Posteriorly, the telson bears two pairs of long, blade-like processes (figure 1*l,n*); each originates adjacent to the midline, tapers to a point, and is laterally flattened and suboval in section. The dorsal processes project posterodorsally at about 30°. The ventral ones curve evenly dorsally through about 60°, their tips crossing immediately outside those of the dorsal pair. There is no evidence for or against mobility in any of these processes. A medial, needle-like process projects posterodorsally from between the ventral pair. The anus lies posteroventrally, as indicated by a faecal stream (figure 1*w,z,a1*). The telson extends parallel to the trunk (figure 1*w*) or may be inclined upwards at about 30° (figure 1*b*).

## 4. Discussion

The preservation of *Enalikter* (figure 1; electronic supplementary material, figure S4) in full three-dimensional form is unique for a stem euarthropod. The trunk of OUMNH C.29632 (figure 1 *m,t–w,y,z,a1*) is subcircular in cross-section, it bends laterally through 180°, and the exopod filaments curve around to hug the bend, in a lowered, presumed ‘in repose’ position (figure 1*t,y*). The other two specimens (figure 1*a,g*) have a flatter trunk section, yet retain upstanding to outstretched limbs, with straight to slightly sinuous, vertically radiating exopod filaments (figure 1*k,o*). Operation of the trunk and filaments by hydraulic pressure might account for such differences of inflation and disposition, though equally it might reflect the early onset of decay.

The pyritized but much larger arthropod (up to 228 mm [30]) *Bundenbachiellus giganteus* [29] (= *Eschenbachiellus wuttkensis* [31]; see [30]) from the Lower Devonian Hunsrück Slate is close in overall morphology to *Enalikter*. Insights from the new Silurian taxon are used here to reinterpret the younger Devonian form. Only one of the two specimens of *Bundenbachiellus* preserves the head ([31], text figures 11–13; electronic supplementary material, figure S3), which was previously interpreted as bearing five appendages. A comparison with the better-preserved *Enalikter* indicates that the structures interpreted by Briggs & Bartels ([31], p. 293) as a uniramous first (evident only on the left side) and a biramous second appendage, together represent a single triflagellate limb. It is likely that the following two (more posterior) head appendages of *Bundenbachiellus* were biramous, although only the endopod is clearly evident (see electronic supplementary material, figure S3). Comparison with the head of *Enalikter* suggests that the appendage interpreted as a fifth head limb in *Bundenbachiellus* may belong to the trunk. There would then be 12 pairs of biramous appendages in the trunk of *Bundenbachiellus* (although their correspondence to tergites is uncertain), as in *Enalikter*, and the posteriormost spines/appendages could be interpreted as telson processes (rather than a pair of spines and a caudal furca) such as those in *Enalikter. Bundenbachiellus* differs from *Enalikter*, however, in a number of ways: the head shield was semicircular (not subrectangular), surrounded by a narrow raised margin; there is no evidence of a whip-like process ventrally on the head; the trunk exopod filaments are leaf-like (not linear) structures with fine spines on their inner margins; and there is no evidence of a medial, needle-like process on the telson. Additionally, the Devonian species is an order of magnitude larger than the Silurian one.

**Figure 2.**
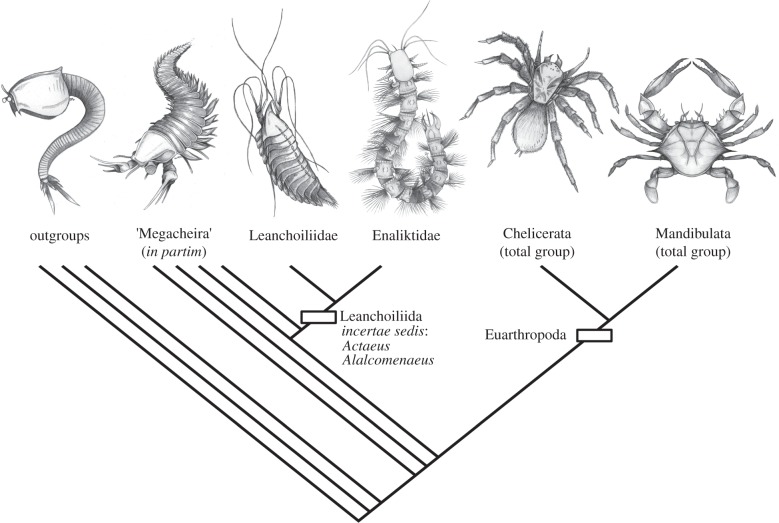
A summary of the phylogenetic relationships and of topologies produced during phylogenetic analyses of Enaliktidae, which were consistent over all of them (see electronic supplementary material, text S1 and figure S1 for details).

*Enalikter* and *Bundenbachiellus* fall in a clade of short-great-appendage (=megacheiran) arthropods [32] that includes *Leanchoilia* from the lower Cambrian of Chengjiang and the middle Cambrian Kaili Lagerstätte, China, and the Burgess Shale, Spence Shale and Marjum Formation of North America; *Alalcomenaeus* from Chengjiang and the Burgess Shale; *Actaeus* from the Burgess Shale; and *Oestokerkus* from the lower Cambrian Emu Bay Shale, Australia [32–37] (figure 2; electronic supplementary material, text S1 and figure S1). Specifically, *Enalikter* is recovered in a clade (Enaliktidae) together with *Bundenbachiellus*. More broadly, it falls under a clade that is the most derived in the euarthropod stem and sister to Euarthropoda, and which also includes the megacheirans *Haikoucaris* and *Parapeytoia* from Chengjiang, and *Yohoia* from Burgess.

Our analysis supports the interpretation of all short-great-appendage arthropods as stem euarthropods [17,22,38–43] rather than as stem chelicerates [18,19,32,44–47].

While the tergopleurae are reduced in some stem euarthropods—for example *Haikoucaris* [18]—enaliktids appear to be unique among stem euarthropods in lacking them entirely. Enaliktids are also distinguished among megacheirans in their lack (loss) of the knuckle/elbow joint between the peduncle and podomeres of the ‘claw’ (flagella), a hallmark of other megacheirans [48] (although this feature is only weakly developed in at least one other purported megacheiran, *Occacaris* [19]). A remarkable feature of *Enalikter* is the long, posteriorly recurved, whip-like anterior process on the head, which may be analogous to the spinose hypostomal structure found in parasitic eucrustaceans [49] (electronic supplementary material, text S2). The ventromedial, subventrally projecting boss-like feature to which the process is attached recalls similar structures interpreted as hypostomal homologues in the stem mandibulates *Agnostus*, *Henningsmoenocaris* and *Martinssonia* [50]; as in those taxa, a discrete, fully sclerotized hypostome is lacking in *Enalikter*. The flat, wide, circumoral disc-like surface in *Enalikter* bears comparison, variously, with the mouth/‘*Peytoia*’ cone of the panarthropod lobopodians *Pamdelurian* and *Opabinia*, and stem euarthropod radiodontids such as *Anomalocaris* and *Peytoia*, and the great appendage arthropod *Parapeytoia* [51–55] (electronic supplementary material, figure S1). In those taxa, however, the oral cone surface is rigid and plated, unlike the disc surface of *Enalikter*, which lacks evidence of plates and was presumed fleshy (see electronic supplementary material, text S2). *Enalikter* inhabited the outer shelf/upper slope of the Anglo-Welsh basin, where water depths might have been up to some 200 m [2]. It is likely to have been a benthic or nektobenthic scavenger/detritivore (see electronic supplementary material, text S2).

Recognition of *Enalikter* and *Bundenbachiellus* in Silurian and Devonian rocks indicates that members of the stem clade Leanchoiliida survived for nearly 100 Myr (75 and 97 Myr, respectively [56]) after the mid-Cambrian *Leanchoilia*? sp. of the Marjum Formation (*ca* 500 Myr BP [34]), the hitherto stratigraphically youngest known short-great-appendage arthropod. *Enalikter* and *Bundenbachiellus* are some 55 and 77 Myr, respectively, younger than the next youngest stem euarthropod, anomalocaridids from the lower Ordovician Fezouta Lagerstätte (*ca* 480 Myr BP) of Morocco [57]; and the enaliktids represent only the second record of stem euarthropods in Silurian or Devonian strata, the other being that of *Schinderhannes* from the Hunsrück Slate [47]. Data on *Enalikter* and *Bundenbachiellus* highlight the importance of rare Silurian and Devonian Konservat–Lagerstätten for revealing the much later, mid- and upper Palaeozoic history of groups such as megacheirans that have previously been considered to be restricted to the Cambrian; more accurate knowledge of their true stratigraphic range is dependent on these critical taphonomic windows. Our study also highlights the advantage available in combining morphological data from different types of exceptional-preservation deposits.

## Data accessibility

Virtual models of the three *Enalikter* specimens in VAXML format (see [21]): Dryad doi:10.5061/dryad.jb0t7. Phylogenetic data matrix in NEXUS format: electronic supplementary material, table S1.
